# Electrochemical oxygen reduction catalysed by Ni_3_(hexaiminotriphenylene)_2_

**DOI:** 10.1038/ncomms10942

**Published:** 2016-03-08

**Authors:** Elise M. Miner, Tomohiro Fukushima, Dennis Sheberla, Lei Sun, Yogesh Surendranath, Mircea Dincă

**Affiliations:** 1 Department of Chemistry, Massachusetts Institute of Technology, 77 Massachusetts Avenue, Cambridge, Massachusetts 02139, USA

## Abstract

Control over the architectural and electronic properties of heterogeneous catalysts poses a major obstacle in the targeted design of active and stable non-platinum group metal electrocatalysts for the oxygen reduction reaction. Here we introduce Ni_3_(HITP)_2_ (HITP=2, 3, 6, 7, 10, 11-hexaiminotriphenylene) as an intrinsically conductive metal-organic framework which functions as a well-defined, tunable oxygen reduction electrocatalyst in alkaline solution. Ni_3_(HITP)_2_ exhibits oxygen reduction activity competitive with the most active non-platinum group metal electrocatalysts and stability during extended polarization. The square planar Ni-N_4_ sites are structurally reminiscent of the highly active and widely studied non-platinum group metal electrocatalysts containing M-N_4_ units. Ni_3_(HITP)_2_ and analogues thereof combine the high crystallinity of metal-organic frameworks, the physical durability and electrical conductivity of graphitic materials, and the diverse yet well-controlled synthetic accessibility of molecular species. Such properties may enable the targeted synthesis and systematic optimization of oxygen reduction electrocatalysts as components of fuel cells and electrolysers for renewable energy applications.

## Abstract (web summary)

There are numerous heterogeneous oxygen reduction reaction catalysts, although synthetic tunability is rare among these materials. Here, the authors report that a conductive metal-organic framework functions as a well-defined, tunable electrocatalyst for the oxygen reduction reaction in alkaline solution.

## Graphical abstract

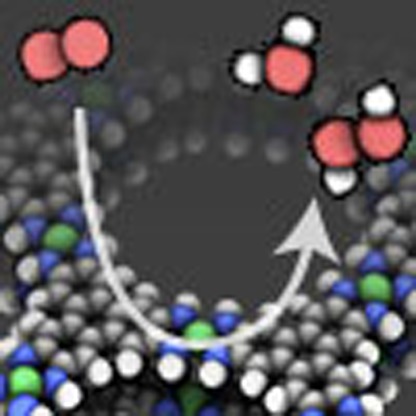

The development of heterogeneous oxygen reduction reaction (ORR) electrocatalysts for implementation into fuel cell and electrolyser cathodes is a major research thrust in the arena of renewable fuel development. Achieving desired architectural and electronic properties of such catalysts remains difficult, however, because several variables must be optimized simultaneously, requiring synthetic tunability that is rarely available in the solid state. Desirable characteristics of an ORR electrocatalyst include: high active site density, reproducible synthesis and catalytic activity, stability in the electrolyte and in oxygen and peroxide, and low overpotential relative to the thermodynamic 4e^−^ oxygen-to-water reduction potential of 1.23 V (versus the reversible hydrogen electrode, RHE). One structural motif that has proven successful in catalysing ORR with high activity and physical robustness is the M-N_*x*_ unit, where M=a non-platinum group metal (for example, Fe, Co, Ni, Cu) chelated in a nitrogenous environment. These structures were popularized after the 1964 report by Jasinski^1^ that detailed the high ORR activity of cobalt phthalocyanine complexes blended with electrically conductive acetylene black. The ability for oxygen to chemisorb onto these M-N_*x*_ sites without degrading the material fuelled extensive investigations of ORR on M-N_*x*_-containing catalysts^2^,^3^,^4^,^5^. Though active towards ORR, M-N_*x*_ complexes have shown inconsistent stability in various electrolytes, motivating high-temperature treatment of the materials to enhance catalyst longevity and electrical conductivity^3^,^5^. Thermal treatment indeed increased the stability of the materials, but introduced new challenges in maintaining synthetic control over structure formation, identifying the catalytic active sites, and establishing structure–function relationships useful for catalyst optimization and mechanistic understanding. Thus, the search for active, intrinsically conductive, and chemically and electrochemically stable ORR electrocatalysts possessing well-defined and tunable active sites continues.

One class of materials that could answer these challenges is metal-organic frameworks (MOFs). These materials are compelling choices for electrocatalytic applications because their high surface area maximizes active site density, and their tunable chemical structure affords tailor-made microenvironments for controllable reaction conditions within the pores. Despite their promising features, MOFs have rarely been used for electrocatalytic applications because they are typically electrical insulators^6^,^7^,^8^,^9^,^10^,^11^. Recently, synthetic advances have given rise to conductive MOFs, some of which exhibit encouraging properties as electrocatalysts^12^,^13^,^14^,^15^,^16^, but to our knowledge none have been experimentally shown to mediate ORR electrocatalysis.

Here we introduce Ni_3_(HITP)_2_ (HITP=2, 3, 6, 7, 10, 11-hexaiminotriphenylene), a conductive two-dimensionally layered material structurally reminiscent of the long-studied M-N_*x*_ ORR electrocatalysts (Fig. 1)^17^, as a representative of a new class of highly ordered ORR electrocatalysts exhibiting ORR activity and electrical conductivity (*σ*=40 S cm^−1^)^17^ with no post-synthetic treatment or modification. In addition to possessing ORR activity competitive with the most active non-platinum group metal (nPGM) electrocatalysts to date, Ni_3_(HITP)_2_ retains 88% of its current density and undergoes no visible morphological degradation during prolonged electrochemical cycling. This study highlights conductive MOFs as a powerful platform for the development of tunable, designer electrocatalysts. It is noted that MOFs have been used as scaffolds for ORR electrocatalysts formed from high-temperature (>600 °C) pyrolysis^18^,^19^,^20^,^21^,^22^,^23^,^24^,^25^,^26^,^27^,^28^,^29^,^30^,^31^,^32^,^33^,^34^,^35^,^36^,^37^ as well as incorporated into composites containing graphene oxide and porphyrin additives^7^,^8^. Whereas such materials indeed exhibit competitive ORR activity, the pyrolysis involved in their preparation eliminates the crystallinity and synthetic control inherent to MOFs. Our aim herein is to introduce a multi-faceted handle on imposing in a controlled manner structural, chemical and electronic properties on our material for reaction-targeted, MOF-based electrocatalyst design.

## Results

### Synthesis and quantification of Ni_3_(HITP)_2_

Ni_3_(HITP)_2_ can be grown solvothermally as a thin film on a variety of electrode surfaces using synthetic conditions mimicking those employed for the synthesis of bulk material^17^. Glassy carbon disk electrodes (5 mm diameter) served as the working electrodes for all investigations described herein unless otherwise noted, and all potentials are referenced to RHE. Deposition of Ni_3_(HITP)_2_ onto the glassy carbon electrodes typically afforded loadings of ∼5 μg of MOF. The loadings were determined precisely in each case by atomic absorption spectroscopy (AAS) and verified by inductively coupled plasma-mass spectrometric (ICP-MS) measurements. The thickness of the film was analysed by atomic force microscopy. Ni_3_(HITP)_2_ films grown on glassy carbon electrodes have a thickness of ∼120 nm, whereas films grown on indium tin oxide exhibit a similar morphology with a thickness of ∼300 nm (Supplementary Fig. 1).

### ORR activity of Ni_3_(HITP)_2_

**Figure 2:**
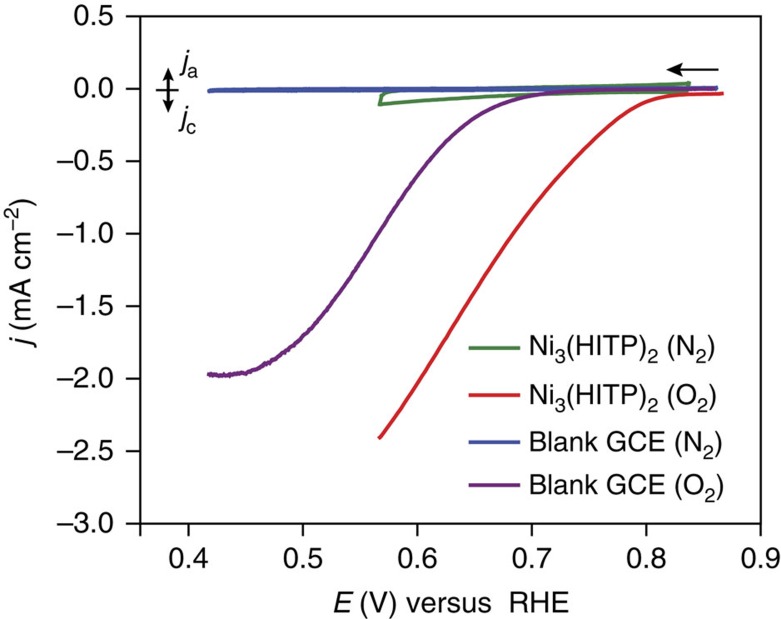
ORR performance. Polarization curves of Ni_3_(HITP)_2_ under N_2_ (green) versus O_2_ atmosphere (red) as well as of the blank glassy carbon electrode under N_2_ versus O_2_ atmosphere (blue and purple, respectively). Scan rate=5 mV s^−1^, rotation rate=2,000 r.p.m., electrolyte=0.10 M aqueous KOH, counter electrode=Pt mesh, reference electrode=Hg/HgO (1.00 M KOH), working electrode=glassy carbon electrode (GCE).

Cyclic voltammograms of Ni_3_(HITP)_2_ thin films on glassy carbon rotating disk electrodes recorded in the absence of O_2_ revealed a significant double layer capacitance that increased with increasing scan rate (Supplementary Fig. 2), reflecting the high surface area of the modified electrodes^38^. Indeed, Ni_3_(HITP)_2_ exhibits a Brunauer–Emmett–Teller-specific surface area of 629.9±0.7 m^2^ g^−1^, as calculated from its nitrogen adsorption isotherm (Supplementary Fig. 3). Under O_2_ atmosphere, the material reduces oxygen with an onset potential (*j*=−50 μA cm^−2^) of 0.82 V in a 0.10 M aqueous solution of KOH (pH=13.0; Fig. 2). The measured ORR onset potential is competitive with the most active nPGM ORR electrocatalysts reported thus far^39^ and sits at an overpotential of 0.18 V relative to Pt (*E*_onset_=1.00 V).

Notably, cyclic voltammetry of the film on indium tin oxide electrodes shows the same ORR activity as the films on the glassy carbon electrodes (Supplementary Fig. 4), verifying that the MOF does not simply enhance the ORR activity of the glassy carbon electrode but rather acts as a stand-alone ORR electrocatalyst regardless of the substrate.

### Stability of Ni_3_(HITP)_2_ during ORR

Steady-state potentiostatic measurements at *E*=0.77 V showed that 88% of the initial current density is retained over 8 h (Supplementary Fig. 5), in-line with other nPGM ORR catalysts^5^,^40^,^41^,^42^,^43^,^44^. Cyclic voltammetry of the modified electrode after the durability study showed no shift in the diffusion-limited region of the polarization curve, indicating that any alterations to the material during electrocatalysis were not significant enough to decrease the mass transport properties of the Ni_3_(HITP)_2_ film (Supplementary Fig. 6). Moreover, cyclic voltammetry of the electrolyte after these 8 h, using a fresh unmodified glassy carbon electrode, indicated no leaching from the Ni_3_(HITP)_2_ films, evidencing the heterogeneous nature of the catalyst. Additionally, ICP-MS and AAS analyses of films before and post electrolysis indicated the same quantity of Ni, suggesting that no part of the catalyst, homogeneous or heterogeneous, is lost from the films during catalysis (Supplementary Tables 1 and 4).

### Characterization of Ni_3_(HITP)_2_ before and after ORR

Spectroscopic, microscopic and diffractometric techniques enabled analysis of the film before and after ORR catalysis. X-ray photoelectron spectroscopy (XPS) of catalyst films before and after catalysis revealed an increase in binding energy of the Ni_2p_ envelope region (850–885 eV) by +1.0 eV (Supplementary Fig. 7). Also visible by XPS was a shoulder peak that was present in the N_1s_ region before ORR (399 eV) which disappears after catalysis (Supplementary Fig. 8). Importantly, though the catalyst may undergo minor structural rearrangement during ORR, the high activity is largely retained over extended steady-state measurements, that is, neither access to the active sites nor the integrity of the active sites themselves is severely compromised during prolonged electrocatalysis. The structural robustness was supported by Raman spectroscopy conducted on the unused film, the film after submersion in the 0.10 M KOH electrolyte, and the film after electrochemical cycling under either N_2_ or O_2_. There were no missing or additional Raman bands for any of the altered films compared with the spectrum of the unused film (Supplementary Fig. 9). Additional evidence supporting the stability of the film was observed in scanning electron micrographs (SEMs) of the film taken before and after ORR catalysis (Supplementary Fig. 10). No perturbations in the morphology of the film were observed upon electrochemical cycling under O_2_. Finally, grazing incidence X-ray diffraction of the Ni_3_(HITP)_2_ film before and after ORR catalysis showed retention of the long-range order in the *ab* plane of Ni_3_(HITP)_2_ during ORR, further highlighting the structural stability of this catalyst during electrochemical cycling under O_2_ (Supplementary Fig. 11).

### ORR kinetics on Ni_3_(HITP)_2_

Using standard rotating ring-disk electrode experiments (Supplementary Fig. 12) and assuming that catalytically competent sites within Ni_3_(HITP)_2_ are distributed homogeneously throughout the film and not just on the surface, lower limit turnover frequencies (TOFs), determined by AAS, were found to be 0.042 electrons [Ni_3_(HITP)_2_]^−1^ s^−1^ and 0.052 electrons [Ni_3_(HITP)_2_]^−1^ s^−1^ for H_2_O_2_ and H_2_O production, respectively, at *E*=0.79 V. Quantifying the Ni content in the same films by ICP-MS gave lower limit TOF values of 0.046 electrons [Ni_3_(HITP)_2_]^−1^ s^−1^ and 0.056 electrons [Ni_3_(HITP)_2_]^−1^ s^−1^ for H_2_O_2_ and H_2_O production, respectively, also at *E*=0.79 V (Supplementary Tables 1–6). The TOF values for H_2_O_2_ and H_2_O production increase by one order of magnitude to 0.491 electrons [Ni_3_(HITP)_2_]^−1^ s^−1^ and 0.466 electrons [Ni_3_(HITP)_2_]^−1^ s^−1^, respectively, at 0.67 V. If the active sites in Ni_3_(HITP)_2_ are the Ni atoms, the lower limit TOFs derived from AAS quantification of Ni are 0.014 electrons [Ni]^−1^ s^−1^ and 0.017 electrons [Ni]^−1^ s^−1^ for H_2_O_2_ and H_2_O production, respectively, at *E*=0.79 V. Minimum TOF values calculated from the ICP-MS quantification of Ni were 0.015 electrons [Ni]^−1^ s^−1^ and 0.019 electrons [Ni]^−1^ s^−1^ for H_2_O_2_ and H_2_O production, respectively, also at *E*=0.79 V. Notably, the intrinsic ORR turnover frequencies for Ni_3_(HITP)_2_ could exceed the values reported here because the Ni quantification methods do not distinguish exclusively electroactive Ni sites; if some fraction of the potentially active sites are not catalytically competent because of mass transport limitations within the films, the ORR current-to-active-site ratio would increase, consequently increasing the TOF.

### Mechanistic insight into ORR on Ni_3_(HITP)_2_

**Figure 3:**
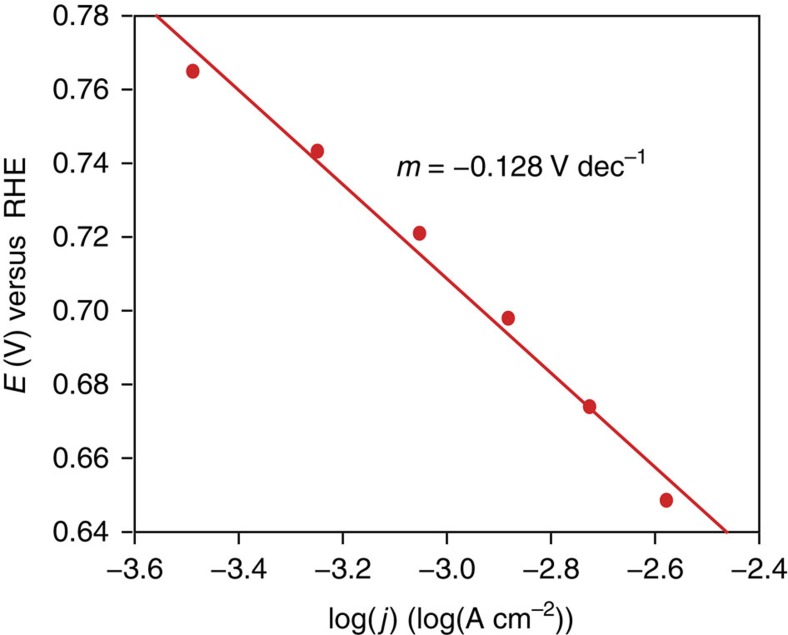
ORR Tafel plot. Activation-controlled Tafel plot for Ni_3_(HITP)_2_-electrocatalyzed ORR, derived from the Koutecky–Levich plots (Supplementary Fig. 13).

The activation-controlled Tafel plot generated from Koutecky–Levich (K–L) data (Supplementary Fig. 13) revealed a Tafel slope of −128 mV dec^−1^ (Fig. 3). This Tafel slope corresponds to an irreversible one-electron pre-equilibrium process, likely indicating the formation of the superoxide anion as the rate-limiting step (theoretical Tafel slope=−120 mV dec^−1^). The total number of electrons transferred during ORR was determined using the inverse of the slope of the K–L plots, termed the *B* factor (equation (1)):


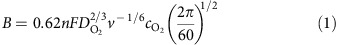


where *n*=number of electrons transferred, *F*=Faraday's constant, 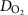
=O_2_ diffusion coefficient in the electrolyte, *v*=kinematic viscosity of the electrolyte, and 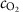
=the saturation concentration of O_2_ in the electrolyte at 1 atm O_2_ pressure. Assuming the typical values in 0.10 M KOH: 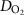
=1.9 × 10^−5^ cm s^−1^, *v*=0.1 m^2^ s^−1^ (ref. 7), and 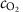
=1.26 × 10^−6^ mol cm^−3^, and using *B*=0.0831, mA cm^−2^ r.p.m.^−1/2^ from the K–L plot at *E*=0.767 V, the number of transferred electrons in our system was calculated to be *n*=2.25 (Supplementary Table 7). This electron transfer number is consistent with predominant (87.5%) production of H_2_O_2_ (more accurately, production of HO_2_^−^ in 0.10 M KOH given the p*K*_a_ of H_2_O_2_=11.63)^45^, with the remaining activity ascribed to 4e^−^ reduction to H_2_O.

**Figure 4:**
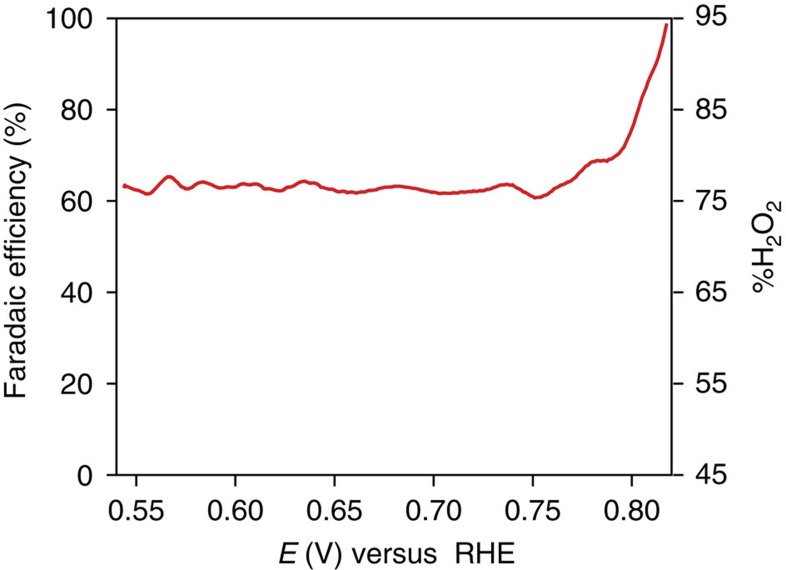
Faradaic efficiency for H_2_O_2_ and %H_2_O_2_. Potential-dependent Faradaic efficiency for H_2_O_2_ production and %H_2_O_2_ production during ORR catalysed by Ni_3_(HITP)_2_ at pH 13.

The Faradaic efficiency for H_2_O_2_ production was determined by measuring the ratio of the ring current to the disk current in rotating ring-disk electrochemical experiments (see Methods section). In the 0.82–0.54 V potential range, the Faradaic efficiency for H_2_O_2_ production decreases from 100 to 63% (Fig. 4) as formation of H_2_O increases with increasing overpotential before reaching a plateau at ∼0.75 V.

The H^+^ order for ORR catalysis was probed galvanostatically at *I*=−5.0 μA in a 0.1 M NaClO_4_/0.1 M NaOH aqueous electrolyte titrated from pH 12.89 to 11.54 with 1.0 M HClO_4_. These studies revealed a slope of zero for δ*E*/δpH above pH 12.80, suggesting a zeroth order dependence on [H^+^] for the kinetic rate law (Supplementary Fig. 14). However, a non-zero slope was observed below pH 12.80, indicating a change in mechanism that involves proton-coupled electron transfer or proton-dependent chemical steps before or during the rate-limiting step (Supplementary Note 1). Detailed mechanistic investigations of ORR with our catalyst are currently underway.

## Discussion

Direct adhesion of the Ni_3_(HITP)_2_ film onto the electrode surface eliminates the need for binders or conductive additives that may block access to active sites by pore filling. This direct contact between the parent material and the electrode allowed for investigation of the inherent electrocatalytic behaviour of pure Ni_3_(HITP)_2_. The high surface area and porosity inherent to Ni_3_(HITP)_2_ may increase the density of and facilitate easy access to the catalytic active sites on Ni_3_(HITP)_2_, contributing to the notable ORR activity. Given that this high ORR activity is observed after the film purification procedure which involves heating the modified electrode in methanol at 65 °C for 20 h, Ni_3_(HITP)_2_ and related materials may be strong candidates for implementation into direct methanol fuel cells where methanol tolerance of the anodic and cathodic catalysts is a necessity^46^. High stability in the presence of methanol is not observed for Pt-based electrocatalysts, a major hurdle currently slowing direct methanol fuel cell development^47^.

Further insight into the robustness of Ni_3_(HITP)_2_ during ORR was achieved using several spectroscopic and microscopic techniques to probe the catalyst structure before and after catalysis. The +1 eV shift in the Ni_2p_ XPS after ORR catalysis could be indicative of a Ni–O interaction^48^,^49^, or alternatively a strengthening of the ligand field as electron density around the imine decreases. The loss of asymmetry in the N_1s_ region of the XPS after catalysis is consistent with an alteration of the ligand field during ORR. Though some minor changes in the film structure may take place during ORR, retention of the majority of ORR activity over the steady-state potentiostatic measurements provides encouraging evidence that neither access to the active sites nor the integrity of the active sites themselves is severely compromised during prolonged electrocatalysis. Furthermore, any subtle alterations of the film affected neither the film's microstructure nor the polarizability of the Ni_3_(HITP)_2_ bonds as shown by SEM and Raman spectroscopy, respectively. The stability of the catalyst in aqueous media is industrially advantageous given the lower cost of water-based electrolytes.

To the best of our knowledge, the foregoing results demonstrate for the first time electrocatalytic ORR activity in a well-defined, intrinsically conductive MOF. Clearly, the faradaic efficiency for water production should be increased for maximizing energy density in industrial settings, but such a goal may be more tractable with MOFs, whose well-defined structures provide the ability to systematically investigate a number of variables including the metal centre identity, valency and coordination environment. Structure–function and mechanistic studies will facilitate understanding, development, and diversification of this material into a platform structure primed for the targeted design of other ORR electrocatalysts.

## Methods

### Characterization of the Ni_3_(HITP)_2_ film

Samples were prepared for ICP-MS and AAS analysis by sonication of the modified electrode buttons in concentrated ICP (Omnitrace purity, 67–70% w/w; EMD) grade nitric acid for 4 h. The electrode buttons were removed from the acid, and the acid was diluted to 2% v/v with Milli-Q water.

ICP-MS was conducted on an Agilent 7900 at the MIT Center for Environmental and Health Sciences (Cambridge, MA, USA). An external calibration curve was generated with a nickel standard (1,000 p.p.m. in 2% HNO_3_; Ultra Scientific) diluted to 0, 15, 30, 60 and 120 p.p.b. in 2% ICP grade nitric acid. Argon flowing at 1.06 l min^−1^ was used as the carrier gas. The ICP-MS data was analysed by MassHunter 4.1 software.

Graphite furnace AAS was conducted on a Perkin Elmer AAnalyst 600 GFAAS (property of the Lippard Group, MIT, Cambridge, MA, USA). An ICP grade Ni standard (1,000 p.p.m. in 2% HNO_3_) (Ultra Scientific) was diluted to 100 p.p.b. in 2% HNO_3_ in Milli-Q water. The AAS performed a serial dilution to generate a nickel calibration curve with 0, 25, 50, 75 and 100 p.p.b. nickel calibration points. The nickel content was probed by monitoring the optical absorption at *λ*=232.0 nm. The graphite furnace temperature was ramped from 110 to 2,500 °C during AAS analysis. The AAS results were analysed by WinLab32 for AA, version 6.5.0.0266.

Atomic force microscopy was conducted at the MIT Institute for Soldier Nanotechnologies (Cambridge, MA, USA) using a Veeco Dimension 3,100 scanning probe microscope (Veeco Digital Instruments by Bruker) equipped with a Nanoscope V controller. Images were recorded in tapping mode in the air at room temperature (23–25 °C) using an Al reflex coated silicon micro cantilever (AC240TS-R3, Asylum Research). The scan rate was set at 1.0 Hz. The atomic force microscopy results were analysed by Gwyddion 2.43 software.

XPS was conducted at the Harvard Center for Nanoscale Systems (Cambridge, MA, USA) on a Thermo Scientific K-Alpha XPS. A survey scan was taken and C, N, O and Ni were probed with a pass energy=50 eV, beam width=400 μm. Data analysis was executed with the Advantage 5.938 software programme.

Raman spectroscopy was conducted on a Horiba Raman spectrophotometer (property of the Myerson Group, MIT, Cambridge, MA, USA) operated at 457 nm with a hole diameter of 500 μm, a slit size of 100 μm, a range of 100–3,000 cm^−1^, a 100 × magnification lens, a laser intensity of 39 A, and 2 s runs with three accumulations per sample.

Scanning electron microscopy was conducted at the Harvard Center for Nanoscale Systems (Cambridge, MA, USA) on a Zeiss Ultra Plus FE-SEM with an InLens detector, a voltage of 10 kV, and 200 k × magnification. Data analysis was executed with SmartSEM V05.04.02.00 software.

Grazing incidence X-ray diffraction was conducted at the MIT Center for Materials Science and Engineering (Cambridge, MA, USA) on a Bruker D8 Discover Diffractometer with a Vantec 2,000 two-dimensional detector, a Cu K_α_ X-ray source (1.5409 Å), and a tube voltage and current of 40 kV and 40 mA, respectively. The diffraction patterns were collected in a grazing incidence geometry with a grazing incidence angle of 3.6°. The blank indium tin oxide slide and the indium tin oxide slides modified with the Ni_3_(HITP)_2_ film were secured onto the diffractometer stage with double-sided tape during data collection. The data for each sample was collected in a single exposure with an exposure time of 10 min per sample. The two-dimensional data were reduced by azimuth averaging over 180° of the Debye Scherrer ring. It is noted that the remaining 180° of the Debye Scherrer ring was blocked by the sample due to the grazing incidence geometry.

### Electrochemistry with the Ni_3_(HITP)_2_ film

KOH (99.99% trace metals) was purchased from Sigma-Aldrich. Oxygen gas was purchased from Airgas (99.8% purity). Reference and glassy carbon working electrodes were purchased from CH Instruments. Pt gauze (100 mesh, 99.9% metal basis) and wires (*φ*=0.404 mm, annealed, 99.9% metal basis, and *φ*=0.5 mm dia., hard, 99.95% metal basis) comprising the auxiliary electrode were purchased from Alfa Aesar. The auxiliary electrode was cleaned by submersion in concentrated HCl followed by sonication for 5 min, washing with Milli-Q water, and drying under a stream of air before each experiment. Working electrodes were cleaned by submersion in concentrated HCl followed by sonication for 5 min, washing with Milli-Q water, and drying under a stream of air. The working electrodes were then sequentially polished with 100, 30 and 5 μm diameter alumina powder from BASI. Unless otherwise noted, all electrochemical experiments were executed with a Bio-Logic SP200 potentiostat/galvanostat in a custom 2-compartment electrochemical cell. Rotating disk electrode and rotating ring-disk electrode studies were conducted with a Bio-Logic VMP3 potentiostat/galvanostat Pine Research Instrumentation Modulated Speed Rotator. Unless otherwise specified, internal resistance of the electrolyte was measured with the Bio-Logic SP200 potentiostat/galvanostat, and iR drop correction was applied. Generally, the resistance of 0.10 M KOH was measured to be ∼40 Ω.

### Synthesis of the Ni_3_(HITP)_2_ film on glassy carbon electrode

2, 3, 6, 7, 10, 11-hexaaminotriphenylene hexahydrochloric acid (HATP·6HCl) salt (10.4 mg) was dissolved in Milli-Q water (6 ml) and heated to 65 °C with stirring in a 20 ml capped glass vial (Vial A). In a second glass reaction vial (Vial B), nickel(II) chloride hexahydrate (4.6 mg) was dissolved in Milli-Q water (4 ml) and to this was added concentrated aqueous ammonium hydroxide (0.4 ml, 25% aqueous solution). The heated HATP solution in Vial A was added to the NiCl_2_/NH_4_OH solution (Vial B) and two alumina micropolished glassy carbon electrodes (5 mm diameter) were placed in the reaction so that the polished faces of the glassy carbon buttons were parallel to the bottom of the reaction vial. Each button was inserted into an NMR tube cap so that only the polished face of the glassy carbon was exposed for modification. The vial was capped and the reaction was heated without stirring at 65 °C for 15 h. The next day, the reaction afforded a translucent film on the glassy carbon electrode buttons. Additionally, a translucent black film was visible on the reaction vial walls and a black flaky solid had settled at the bottom of the reaction vial. The electrode film and the reaction mixture solid were purified separately.

The electrode was removed from the reaction mixture and heated in Milli-Q water (20 ml) at 65 °C for 4 h in a capped vial, rinsed with Milli-Q water, then heated again in water at 65 °C for 15 h in a capped vial. The electrode was rinsed with CH_3_OH and then heated in fresh CH_3_OH in a capped vial at 65 °C for 5 h. The CH_3_OH was removed and the electrode was heated at 65 °C for 15 h in fresh CH_3_OH. The next day after drying under dynamic vacuum, a black translucent film coating the polished side of the glassy carbon button was visible. The electrode button was stored under dynamic vacuum.

For purification of the black powder, the remaining reaction mixture was centrifuged, the supernatant was removed, and the remaining solid was sonicated in Milli-Q water (15 ml) for 5 min then heated in a capped vial with stirring at 65 °C for 4 h. The same procedure was repeated once more, with the final heating step duration of 15 h. The powder was once again centrifuged, followed by removal of the supernatant, and then the powder was sonicated in CH_3_OH (15 ml) for 5 min, then heated in the capped vial in CH_3_OH at 65 °C for 5 h. The CH_3_OH wash procedure was also repeated one more time, then the powder was centrifuged, the supernatant was removed, and the black solid was dried under vacuum for 15 h.

### Determination of the 2-and-4-electron ORR TOFs

The background-subtracted ring current (Supplementary Fig. 12) was taken for each potential probed during potentiostatic measurements (*E*_disk_=0.807, 0.787, 0.767, 0.747, 0.727, 0.707, 0.687, and 0.667 V) then divided by 1,000 to calculate current passed in A=C s^−1^ (*I*=*Q*/*t*). That current was divided by 0.2 to account for the 20% ring collection efficiency, then divided by Faraday's constant (96,485.3365 C mol^−1^) and multiplied by Avogadro's number (6.022 × 10^23^ electrons per mol) to determine the number of electrons transferred to O_2_ when reducing O_2_ to H_2_O_2_ (2-electron ORR) per second. By ICP-MS the electrode was calculated to have an average of 1.1015 × 10^16^ nickel sites deposited (see Supplementary Table 1 for s.d.). By AAS, the electrode was calculated to have an average of 1.26199 × 10^16^ nickel sites deposited (see Supplementary Table 4 for s.d.). The number of electrons transferred per second was divided by the number of nickel sites as determined by AAS and ICP-MS, respectively, to convert the 2-electron ORR TOF to electrons nickel per site per second according to the two nickel quantification methods (that is, AAS and ICP-MS) (Supplementary Tables 2 and 5). Alternatively, the number of electrons transferred per second was divided by the number of Ni_3_(HITP)_2_ units to convert the 2-electron ORR TOF to electrons per Ni_3_(HITP)_2_ formula unit per second. The number of Ni_3_(HITP)_2_ formula units was directly calculated from the number of nickel sites derived from the two nickel quantification methods. In the main text, the TOF is expressed as a range defined by the values calculated using the two employed nickel quantification methods.

To determine the TOF for 4-electron ORR, the background and collection efficiency-corrected ring current was subtracted from the disk current (A) (Supplementary Fig. 12) to obtain the current passed during 4-electron ORR. The current (A) was divided by Faraday's constant then by number of nickel or Ni_3_(HITP)_2_ sites to calculate the TOF (electrons per nickel site per second, or electrons per Ni_3_(HITP)_2_ formula unit per second, respectively) during 4-electron ORR according to the two employed nickel quantification methods (Supplementary Tables 3 and 6). In the main text, the TOF is expressed as a range defined by the values calculated using the two employed nickel quantification methods.

### Rotating disk and rotating ring-disk electrode investigations

Experiments were conducted in a two-compartment cell with a glass frit separating the auxiliary electrode from the working electrode; electrolyte=0.10 M KOH; auxiliary electrode=Pt mesh; reference electrode=Hg/HgO (1.00 M KOH), working electrode=blank glassy carbon button (5 mm diameter) or glassy carbon button modified with Ni_3_(HITP)_2_ film and inserted in a polyarylether-ether ketone rotating ring-disk electrode (RRDE) tip with a platinum ring; rotation rate=2,000 r.p.m.; scan speed=5 mV s^−1^; atmosphere=N_2_ or O_2_ sparged for 10 min through a fritted sparge tube before data collection, with continuous sparging during data collection. All voltammograms were collected by scanning cathodically from *E*=0 V versus open circuit potential (OCP) to −0.300 V versus Hg/HgO (0.567 V versus RHE). The electrolyte solvent window was established by cycling a blank glassy carbon button under N_2_ atmosphere. ORR activity of the unmodified glassy carbon was observed by cycling the unmodified glassy carbon electrode under O_2_ from *E*=0 V versus OCP to *E*=0.400 V versus RHE. These controls preceded data collection of Ni_3_(HITP)_2_-modified glassy carbon under N_2_ and O_2_ atm. When relevant, a potential of *E*=1.23 V versus RHE was applied to the Pt ring disk for oxidation of the ORR products. A 20% collection efficiency was applied for quantification of the ORR products using the current measured at the Pt ring disk.

### Potentiostatic steady-state durability test

While rotating at 2,000 r.p.m., cyclic voltammetry (CV) of the Ni_3_(HITP)_2_-modified glassy carbon electrode was conducted at 5 mV s^−1^ from *E*=0 V versus OCP to *E*=−0.3 V versus Hg/HgO (*E*=0.567 V versus RHE) under sparging O_2_ atmosphere to measure the ORR activity. In this experiment, a titanium plate auxiliary electrode was used. O_2_ sparged throughout the entirety of the experiment. The potential was held at *E*=0.767 V versus RHE for 8 h with O_2_ sparging continuously. The current response was monitored with data points collected every 60 s. After the potentiostatic stability test was completed, CV was conducted again under O_2_ atmosphere at 5 mV s^−1^ from *E*=0 V versus OCP to *E*=−0.3 V versus Hg/HgO to compare the mass transport of the used material to that of the material before the stability test.

### Koutecky-Levich and Tafel studies

CV (5 mV s^−1^) under N_2_ atmosphere was conducted from 0 V versus OCP to −0.3 V versus Hg/HgO (ORR potential range for Ni_3_(HITP)_2_). CV (5 mV s^−1^) under O_2_ atmosphere was conducted from 0 V versus OCP to −0.3 V versus Hg/HgO (ORR potential range for Ni_3_(HITP)_2_). Galvanostatic measurements were conducted with *I*=−1, −10 and −100 μA to identify the reliable potential range for potentiostatic measurements. Potentiostatic measurements were conducted from −20 to −200 mV versus Hg/HgO in increments of 20 mV. Each potential was held for 1 min. This was conducted five times, with altering rotation speeds to extrapolate the diffusion coefficient. The electrode was rotated at 2,000, 625, 816, 550 and 1,189 r.p.m., respectively. This allowed for elimination of mass transport limitations when analysing Tafel behaviour via generation of the activation-controlled Tafel plot. CV (5 mV s^−1^) under O_2_ atmosphere was conducted from 0 V versus OCP to −0.3 V versus Hg/HgO (ORR potential range for Ni_3_(HITP)_2_). Chronoamperometry at *E*=−0.2 V versus Hg/HgO was run for 8 min under N_2_ sparging atmosphere to eliminate O_2_. CV (5 mV s^−1^) under N_2_ atmosphere was conducted from 0 V versus OCP to −0.3 V versus Hg/HgO (ORR potential range for Ni_3_(HITP)_2_) to recheck the double layer capacitance as an indicator of potential catalyst decomposition. Ohmic drop was measured at *I*=−0.1 mA for iR correction.

### ORR proton order study

Potential was measured over 25 min at a constant current *I*=−5 μA while varying the pH from 12.89 to 11.54 in the 0.10 M KOH electrolyte titrated with 1.0 M HClO_4_.

## Additional information

**How to cite this article:** Miner, E. M. *et al.* Electrochemical oxygen reduction catalysed by Ni_3_(hexaiminotriphenylene)_2_. *Nat. Commun.* 7:10942 doi: 10.1038/ncomms10942 (2016).

## Supplementary Material

Supplementary Information Supplementary Figures 1-13, Supplementary Tables 1-7, Supplementary Note 1 and Supplementary References

## Figures and Tables

**Figure 1:**
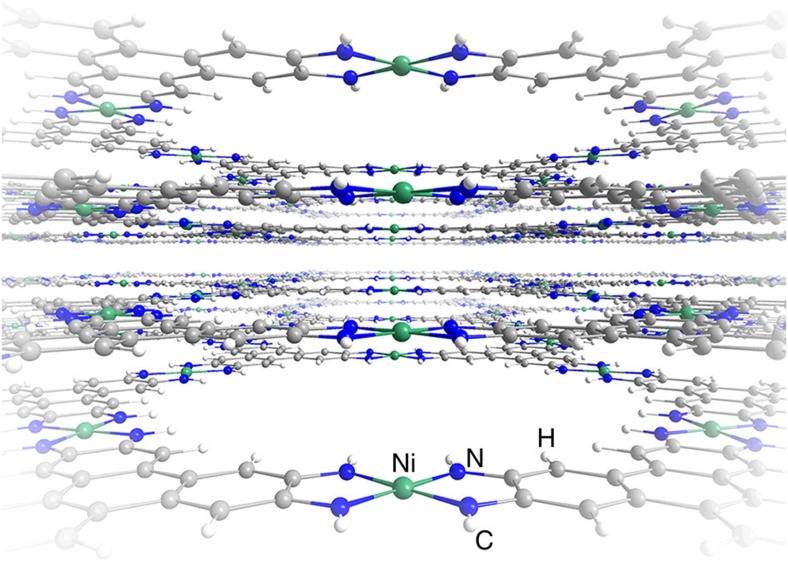
Ni_3_(HITP)_2_ structure. Perspective view of the two-dimensional layered structure of Ni_3_(HITP)_2_ (ref. 17).
