# Supplementary material for: Electrochemical oxygen reduction catalysed by Ni3(hexaiminotriphenylene)2
Source: Nat Commun. 2016 Mar 8;7:10942. doi: 10.1038/ncomms10942 (PMC4786780; doi:10.1038/ncomms10942)
Supplement: Supplementary Information — Supplementary Figures 1-13, Supplementary Tables 1-7, Supplementary Note 1 and Supplementary References [file ncomms10942-s1.pdf]

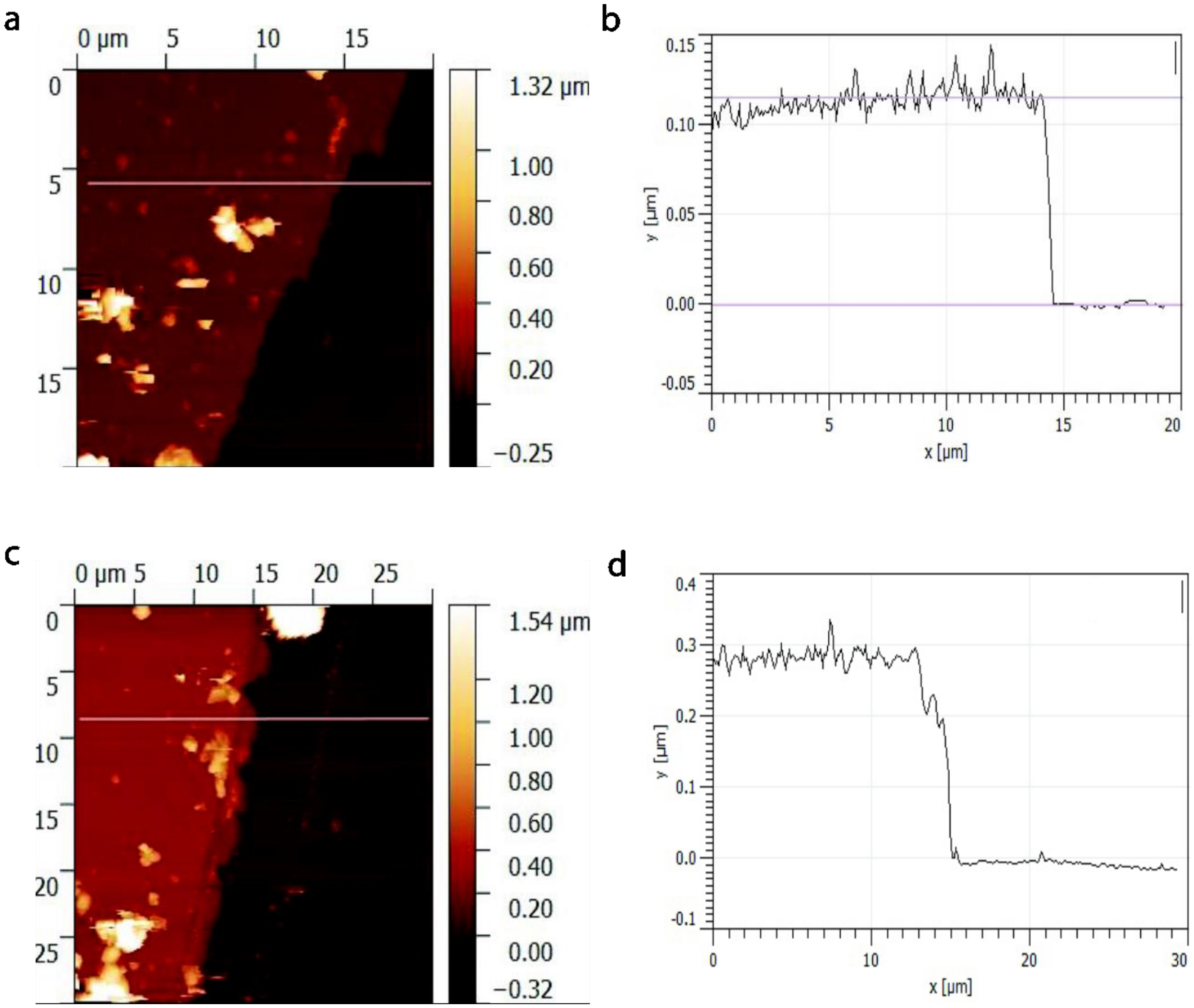

Supplementary Figure 1 AFM measurements. a) Thickness / roughness and **b)** height profile of $Ni_3(HITP)_2$ film on a GC electrode, and **c)** thickness / roughness and **d)** height profile of $Ni_3(HITP)_2$ film on an ITO electrode as measured by atomic force microscopy.

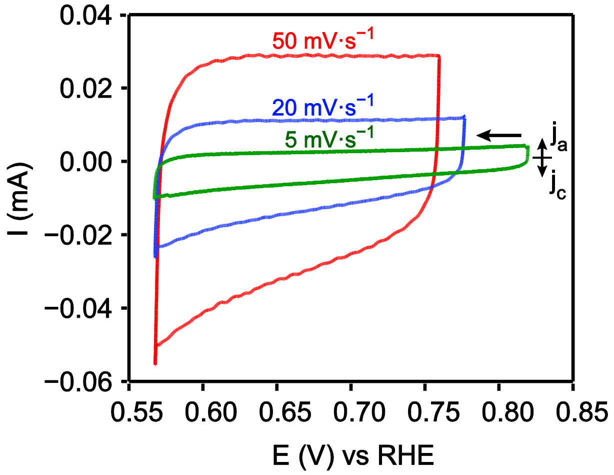

Supplementary Figure 2 Double layer capacitance. Double layer capacitance polarization curves of $Ni_3(HITP)_2$ under N_2 atmosphere with varying CV scan rates. The initial potential shift among the three scans is a result of the working electrode open circuit potential shifting during electrolysis.

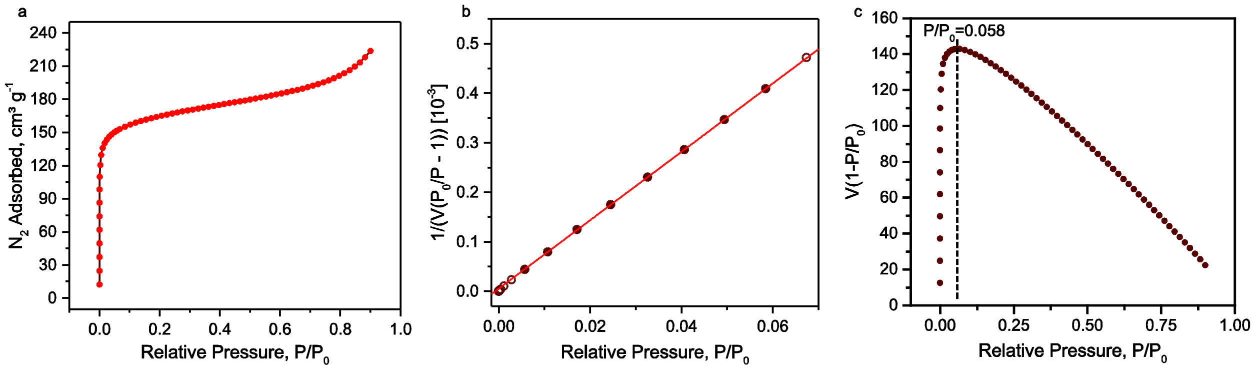

Supplementary Figure 3 BET surface area. Nitrogen adsorption isotherm for $Ni_3(HITP)_2$ powder at 77 K. **a**) adsorption isotherm, **b**) BET linear fit, **c**) $V(1-P/P_0)$ vs. P/P_0 plot used to select upper limit for BET linear fit. Range selection and consistency checks for BET fit were done as suggested by Rouquerol et al.[1]

Relevant data for BET fit of the N_2 adsorption isotherm

BET Surface Area	629.9 ± 0.7 m^2 g^{-1}
C	1253
Correlation Coefficient	0.9999962
Fit range (P/P_0)	0.0057-0.0585 (8 points)
V_m	144.7 cm^3 g^{-1} STP
P/P_0 @ V_m	0.0282
$1/(\sqrt{C}+1)$	0.0275

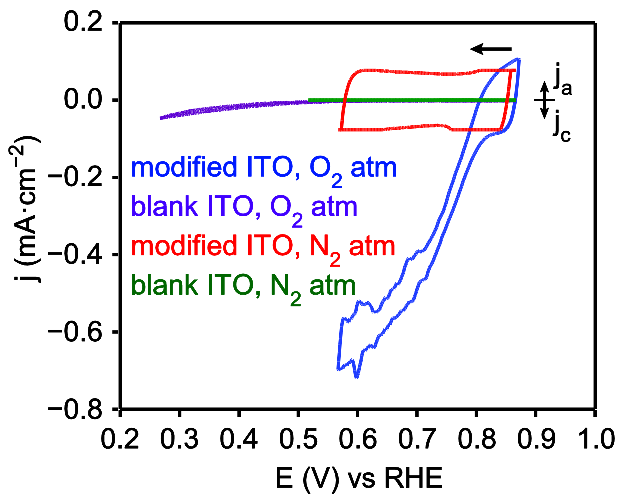

Supplementary Figure 4 ORR on modified ITO. Cyclic voltammograms of $Ni_3(HITP)_2$-modified and blank indium tin oxide (ITO) electrodes under N_2 and O_2 atmosphere. Fluctuations in the polarization curve of the $Ni_3(HITP)_2$-modified ITO (blue trace) are due to interference from sparging O_2 gas during data collection. The hysteresis observed in the $Ni_3(HITP)_2$ polarization curve under O_2 atmosphere is ascribed to pseudocapacitance.

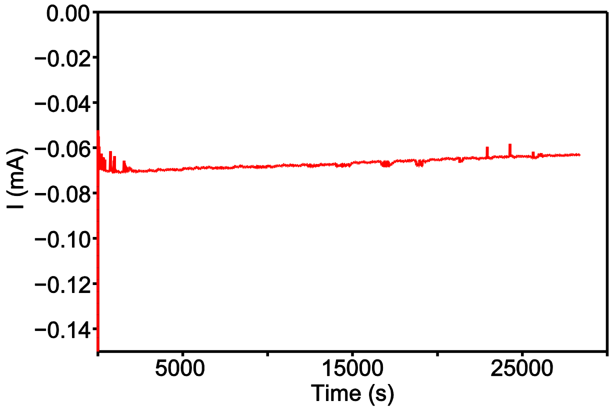

Supplementary Figure 5 Potentiostatic stability test. Potentiostatic stability test of $Ni_3(HITP)_2$ electrocatalyzing ORR at E = 0.767 V versus RHE over 8 hours. It is noted that the periodic current spikes were concomitant with spikes in the potential applied to the disk, presumably due to some disturbance in the contact of the rotator to the electrode shaft. The slight fluctuations in potential never exceeded the potential range in which $Ni_3(HITP)_2$ is stable, so potential changes likely did not influence the catalyst stability in this experiment.

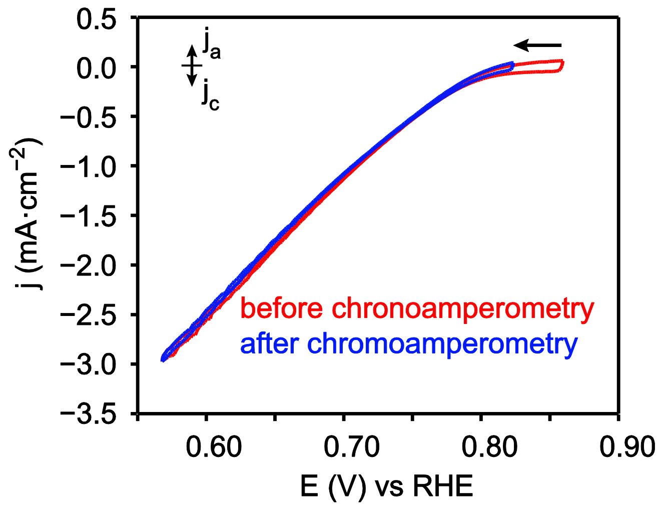

Supplementary Figure 6 Stability after cycling under O_2. Cyclic voltammograms of $Ni_3(HITP)_2$ before and after the potentiostatic stability test shown in Supplementary Figure 5, under O_2 atmosphere.

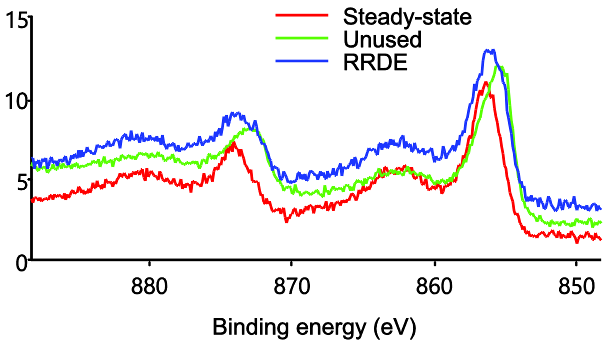

Supplementary Figure 7 Ni_{2p} XPS. X-ray photoelectron spectrum of $Ni_3(HITP)_2$ film on GCE Ni_{2p} envelope region. Both unused (before ORR) and used (after ORR) samples are shown here.

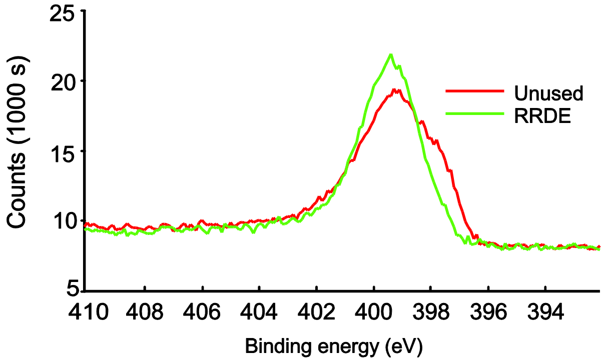

Supplementary Figure 8 N_{1s} XPS. X-ray photoelectron spectrum of $Ni_3(HITP)_2$ film on GCE N_{1s} envelope region. Both unused (before ORR) and used (after ORR) samples are shown here.

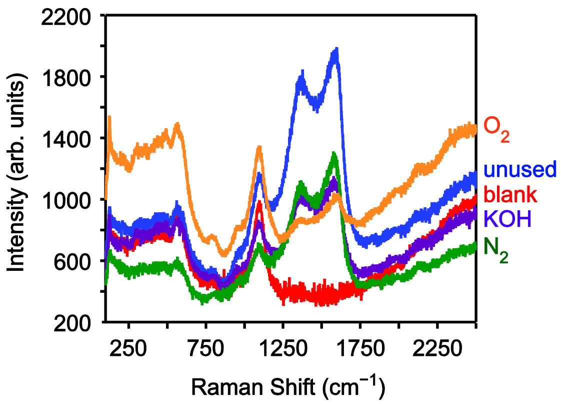

Supplementary Figure 9 Raman spectra. Raman spectra of $Ni_3(HITP)_2$ on ITO electrodes. The blank ITO electrode (blank), the unused $Ni_3(HITP)_2$-modified electrode (unused), the Ni3(HITP)2-modified electrode submerged in the 0.10 M KOH electrolyte for 1 hour (KOH), the $Ni_3(HITP)_2$-modified electrode cycled exclusively under N_2 atmosphere (N_2), and the $Ni_3(HITP)_2$-modified electrode cycled exclusively under O_2 atmosphere (O_2) are shown in red, blue, purple, green, and orange respectively.

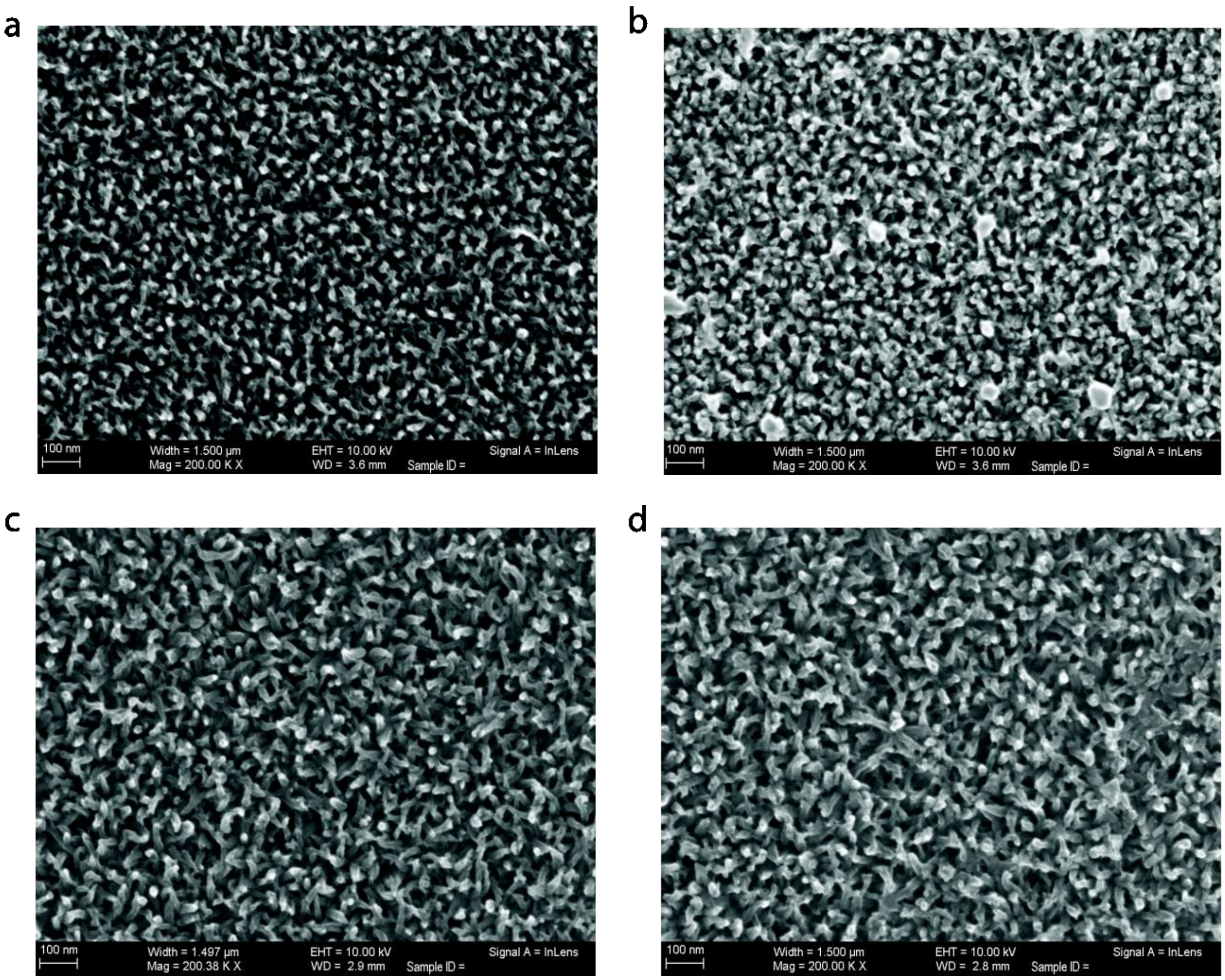

Supplementary Figure 10 SEM images. Scanning electron micrographs of $Ni_3(HITP)_2$ **a-b)** on GC electrodes and **c-d)** on ITO electrodes **a, c)** before ORR catalysis versus **b, d)** after ORR catalysis.

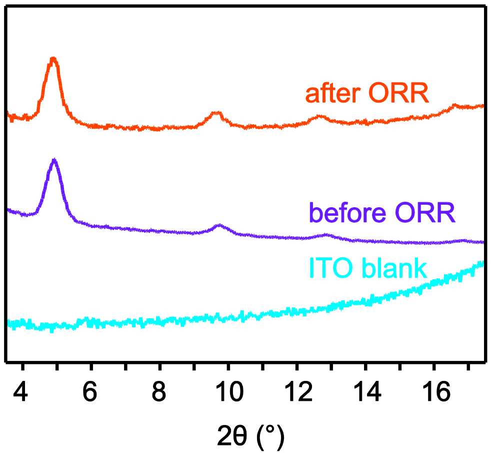

Supplementary Figure 11 Grazing incidence X-ray diffraction patterns. Diffraction patterns of unmodified ITO (teal), $Ni_3(HITP)_2$ film on ITO before ORR (purple), and $Ni_3(HITP)_2$ film on ITO after ORR (orange).

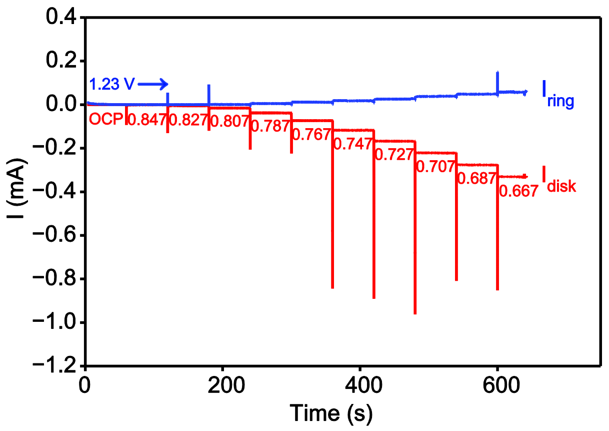

Supplementary Figure 12 Potentiostatic RRDE data. Potentiostatic rotating ring disk electrode measurement for determination of 2e⁻ and 4e⁻ ORR TOF as catalyzed by $Ni_3(HITP)_2$. Disk potentials (red) are referenced to RHE. A constant potential of 1.23 V versus RHE was applied to the platinum ring (blue).

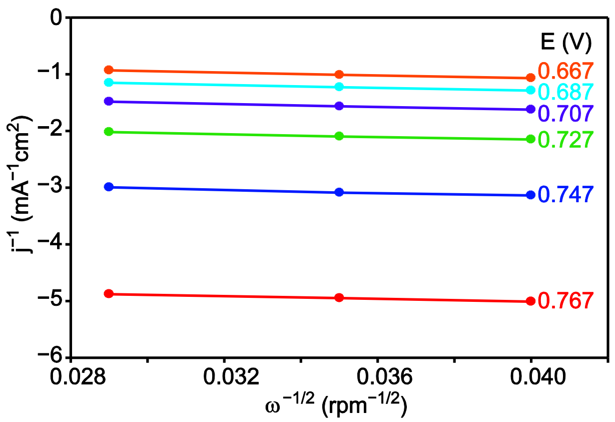

Supplementary Figure 13 Koutecky-Levich plots. Koutecky-Levich plots from $Ni_3(HITP)_2$-electrocatalyzed ORR. See below for electron transfer numbers derived from K-L B factors.

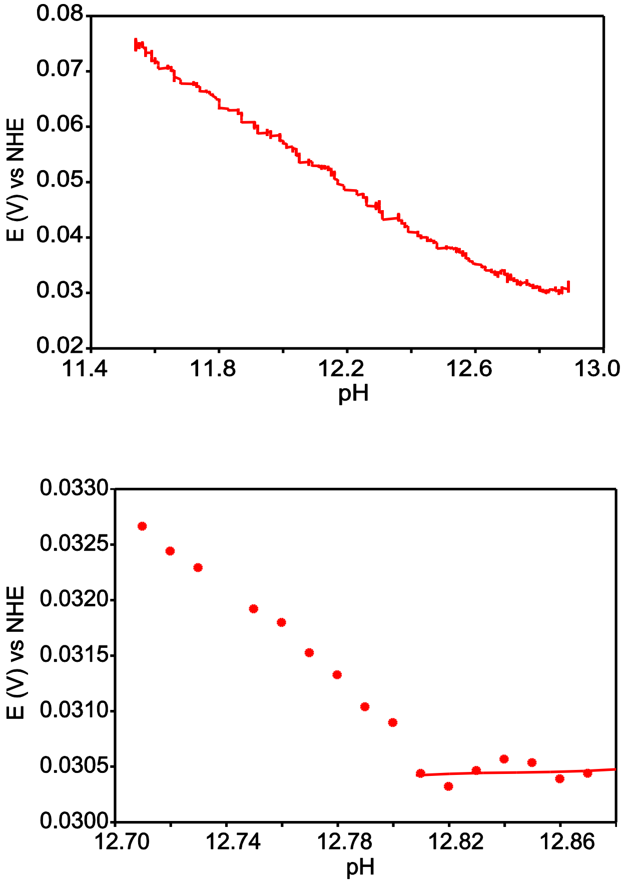

Supplementary Figure 14 [H^+] order data. Top: Galvanostatic ORR proton order study at $I = -5$ μA, pH 11.54-12.89. Bottom: Magnified portion of the plot for the galvanostatic ORR proton order experiment highlighting the inflection point at pH 12.80, which indicates a deviation from a zeroth order in [H^+] dependence.

Supplementary Table 1 ICP-MS quantification of Ni. Calculated number of nickel sites from ICP-MS quantification of nickel sites on glassy carbon electrodes

Sample	$[Ni]_{avg}$ (ppb)	SD	# Ni sites on electrode	Catalyst loading (μg $Ni_3(HITP)_2$)	mol $Ni_3(HITP)_2$
RRDE	58.393	3.562	$1.198 \cdot 10^{16}$	5.311	$6.633 \cdot 10^{-9}$
unused	47.147	3.006	$9.675 \cdot 10^{15}$	4.288	$5.355 \cdot 10^{-9}$
unused	55.053	5.043	$1.130 \cdot 10^{15}$	5.008	$6.253 \cdot 10^{-9}$

Supplementary Table 2 $2e^-$ ORR TOF calculations. $2e^-$ ORR TOF calculations using nickel loading determined by ICP-MS

E (V) vs RHE	background and collection efficiency corrected I_{ring} (A)	$2e^-$ ORR TOF (electrons·s^{-1})	$2e^-$ ORR TOF (electrons·$[Ni_3(HITP)_2]^{-1}$·s^{-1})	$2e^-$ ORR TOF (electrons·$[Ni]^{-1}$·s^{-1})
0.807	$3.496 \cdot 10^{-6}$	$2.182 \cdot 10^{13}$	0.00596	0.00199
0.787	$2.674 \cdot 10^{-5}$	$1.669 \cdot 10^{14}$	0.0456	0.0152
0.767	$5.456 \cdot 10^{-5}$	$3.405 \cdot 10^{14}$	0.0930	0.0320
0.747	$8.367 \cdot 10^{-5}$	$5.222 \cdot 10^{14}$	0.143	0.0475
0.727	0.000136	$8.498 \cdot 10^{14}$	0.232	0.0774
0.707	0.000192	$1.198 \cdot 10^{14}$	0.327	0.109
0.687	0.000242	$1.508 \cdot 10^{14}$	0.412	0.137
0.667	0.000288	$1.798 \cdot 10^{14}$	0.491	0.164

Supplementary Table 3 $4e^-$ ORR TOF calculations. $4e^-$ ORR TOF calculations using nickel loading determined by ICP-MS

E (V) vs RHE	I_{disk} − corrected I_{ring} (A)	$4e^-$ ORR TOF (electrons·s^{-1})	$4e^-$ ORR TOF (electrons·$[Ni_3(HITP)_2]^{-1}$·s^{-1})	$4e^-$ ORR TOF (electrons·$[Ni]^{-1}$·s^{-1})
0.807	$1.539 \cdot 10^{-5}$	$9.608 \cdot 10^{13}$	0.0262	0.00875
0.787	$3.309 \cdot 10^{-5}$	$2.066 \cdot 10^{14}$	0.0564	0.0188
0.767	$6.288 \cdot 10^{-5}$	$3.924 \cdot 10^{14}$	0.107	0.0357
0.747	0.000101	$6.329 \cdot 10^{14}$	0.173	0.0576
0.727	0.000141	$8.800 \cdot 10^{14}$	0.240	0.0801
0.707	0.000184	$1.148 \cdot 10^{15}$	0.313	0.104
0.687	0.000229	$1.430 \cdot 10^{15}$	0.390	0.130
0.667	0.000273	$1.706 \cdot 10^{15}$	0.466	0.155

Supplementary Table 4 AAS quantification of Ni. AAS quantification of nickel sites on glassy carbon electrodes

Sample	$[Ni]_{avg}$ (ppb)	SD	%RSD	# Ni sites on electrode	Catalyst loading (μg $Ni_3(HITP)_2$)	mol $Ni_3(HITP)_2$
RRDE	61.92	0.570	0.921	$1.26199 \cdot 10^{16}$	5.6321	$7.03316 \cdot 10^{-9}$
unused	51.73	0.414	0.801	$1.06151 \cdot 10^{16}$	4.70523	$5.8757 \cdot 10^{-9}$
unused	59.47	0.831	1.397	$1.22034 \cdot 10^{16}$	5.40924	$6.75488 \cdot 10^{-9}$

Supplementary Table 5 $2e^-$ ORR TOF calculations. $2e^-$ ORR TOF calculations using nickel loading determined by AAS

E (V) vs RHE	background and collection efficiency corrected I_{ring} (A)	$2e^-$ ORR TOF (electrons·s^{-1})	$2e^-$ ORR TOF (electrons·$[Ni_3(HITP)_2]^{-1}$·s^{-1})	$2e^-$ ORR TOF (electrons·$[Ni]^{-1}$·s^{-1})
0.807	$3.496 \cdot 10^{-6}$	$2.182 \cdot 10^{13}$	0.0554	0.00185
0.787	$2.674 \cdot 10^{-5}$	$1.669 \cdot 10^{14}$	0.0424	0.0141
0.767	$5.456 \cdot 10^{-5}$	$3.405 \cdot 10^{14}$	0.0865	0.0288
0.747	$8.367 \cdot 10^{-5}$	$5.222 \cdot 10^{14}$	0.133	0.0442
0.727	0.000136	$8.498 \cdot 10^{14}$	0.216	0.0719
0.707	0.000192	$1.198 \cdot 10^{15}$	0.304	0.101
0.687	0.000242	$1.508 \cdot 10^{15}$	0.383	0.128
0.667	0.000288	$1.798 \cdot 10^{15}$	0.457	0.152

Supplementary Table 6 $4e^-$ ORR TOF calculations. $4e^-$ ORR TOF calculations using nickel loading determined by AAS

E (V) vs RHE	I_{disk} − corrected I_{ring} (A)	$4e^-$ ORR TOF (electrons·s^{-1})	$4e^-$ ORR TOF electrons·$[Ni_3(HITP)_2]^{-1}$·s^{-1})	$4e^-$ ORR TOF electrons·$[Ni]^{-1}$·s^{-1})
0.807	$1.539 \cdot 10^{-5}$	$9.608 \cdot 10^{13}$	0.0244	0.00813
0.787	$3.310 \cdot 10^{-5}$	$2.066 \cdot 10^{14}$	0.0525	0.0175
0.767	$6.288 \cdot 10^{-5}$	$3.924 \cdot 10^{14}$	0.0997	0.0332
0.747	0.000101	$6.329 \cdot 10^{14}$	0.161	0.0536
0.727	0.000141	$8.780 \cdot 10^{14}$	0.223	0.0745
0.707	0.000184	$1.148 \cdot 10^{14}$	0.291	0.0972
0.687	0.000229	$1.430 \cdot 10^{15}$	0.363	0.121
0.667	0.000273	$1.706 \cdot 10^{15}$	0.433	0.144

Determination of electron transfer number n from K-L plots:

The following equation relates the inverse of the slope of the K-L plots, B (in $A \cdot cm^{-2} \cdot rpm^{-0.5}$):[2]

$$B = 0.62nFD_{O_2}{}^{2/3}v^{-1/6}c_{O_2}(\frac{2\pi}{60})^{1/2}$$

n = number of electrons transferred

F = Faraday's constant (96485 $C \cdot mol^{-1}$)

D_{O_2} = diffusion coefficient of O_2 in 0.1 M KOH ($1.93 \cdot 10^{-5}$ $cm^2 \cdot s^{-1}$)[1]

v = kinematic viscosity of 0.1 M KOH ($1.09 \cdot 10^{-2}$ $cm^2 \cdot s^{-1}$)[1]

c_{O_2} = saturation concentration of O_2 in 0.1 M KOH at 1 atm O_2 pressure ($1.26 \cdot 10^{-6}$ $mol \cdot cm^{-3}$)[1]

Supplementary Table 7 Electron transfer numbers. Electron transfer calculations from K-L plots at varying potentials

***E* (V) vs RHE**	***B* ($A \cdot cm^{-2} \cdot rpm^{-0.5}$)**	***n* (electrons)**
0.767	$-8.391 \cdot 10^{-5}$	2.25
0.747	$-7.590 \cdot 10^{-5}$	2.04
0.727	$-7.669 \cdot 10^{-5}$	2.06
0.707	$-7.625 \cdot 10^{-5}$	2.05
0.687	$-7.920 \cdot 10^{-5}$	2.12
0.667	$-8.315 \cdot 10^{-5}$	2.23

Supplementary Notes

Supplementary Note 1. The observed potential dependence on $[H^+]$ is unlikely related to the relative ionic strengths of the titrated electrolytes, which could influence the rate of O_2 uptake in the pores as well as the electron mobility; the system was sparged with O_2 for 10 minutes prior to electrolysis.

Supplementary References

1. Rouquerol, J.; Rouquerol, F.; Llewellyn, P.; Maurin, G.; Sing, K. S. W. *Adsorption by Powders and Porous Solids: Principles, Methodology and Applications*. (Academic Press: London, 2013).

2. Min, X., Chen, Y. & Kanan, M. W. Alkaline O_2 reduction on oxide-derived Au: high activity and 4e− selectivity without (100) facets. *Phys. Chem. Chem. Phys.* **16,** 13601 (2014).
